# The Rationale for Potential Pharmacotherapy of COVID-19

**DOI:** 10.3390/ph13050096

**Published:** 2020-05-14

**Authors:** Maha Saber-Ayad, Mohamed A. Saleh, Eman Abu-Gharbieh

**Affiliations:** 1 Department of Clinical Sciences, College of Medicine, University of Sharjah, Sharjah 27272, UAE; mohamed.saleh@sharjah.ac.ae (M.A.S.); eabugharbieh@sharjah.ac.ae (E.A.-G.); 2 College of Medicine, Cairo University, Cairo 12613, Egypt; 3 Department of Pharmacology and Toxicology, Faculty of Pharmacy, Mansoura University, Mansoura 35516, Egypt

**Keywords:** COVID-19, SARS-CoV-2, remdesivir, chloroquine, lopinavir, favipiravir, baricitinib, interferons, ACE2, TMPRSS2

## Abstract

On 11 March 2020, the coronavirus disease (COVID-19) was defined by the World Health Organization as a pandemic. Severe acute respiratory syndrome-2 (SARS-CoV-2) is the newly evolving human coronavirus infection that causes COVID-19, and it first appeared in Wuhan, China in December 2019 and spread rapidly all over the world. COVID-19 is being increasingly investigated through virology, epidemiology, and clinical management strategies. There is currently no established consensus on the standard of care in the pharmacological treatment of COVID-19 patients. However, certain medications suggested for other diseases have been shown to be potentially effective for treating this infection, though there has yet to be clear evidence. Therapies include new agents that are currently tested in several clinical trials, in addition to other medications that have been repurposed as antiviral and immune-modulating therapies. Previous high-morbidity human coronavirus epidemics such as the 2003 SARS-CoV and the 2012 Middle East respiratory syndrome coronavirus (MERS-CoV) prompted the identification of compounds that could theoretically be active against the emerging coronavirus SARS-CoV-2. Moreover, advances in molecular biology techniques and computational analysis have allowed for the better recognition of the virus structure and the quicker screening of chemical libraries to suggest potential therapies. This review aims to summarize rationalized pharmacotherapy considerations in COVID-19 patients in order to serve as a tool for health care professionals at the forefront of clinical care during this pandemic. All the reviewed therapies require either additional drug development or randomized large-scale clinical trials to be justified for clinical use.

## 1. Introduction

The pandemic of the coronavirus disease (COVID-19) has quickly spread to affect almost all countries and territories around the world. The agent causing the infection was rapidly detected to be a beta coronavirus, first named the novel coronavirus (2019-nCoV). The International Committee of Taxonomy of Viruses changed the name to severe acute respiratory syndrome-2 (SARS-CoV-2), denoting the coronavirus as causing severe acute respiratory syndrome-2 [1].

The reported COVID-19 case fatality ranges from 1% to 7%. According to reports of the World Health Organization (WHO), these values must be interpreted with caution. In countries that have implemented massive screening for the whole population, like South Korea, the overall case fatality may be less than 1%, as a large number of mild or asymptomatic cases have been counted in the denominator of their equation. Due to the huge, unprecedented scale of the pandemic, the actual numbers of deaths are considerable [2].

Respiratory failure was obviously a major cause of death in previous viral pandemics, including Spanish flu in 1918 and Middle East respiratory syndrome coronavirus (MERS-CoV) in 2012, and this is also the case in the new COVID-19 [3]. Besides conventional treatment, antiviral therapy (both classic and novel repurposed medications) may shorten the course of the disease and improve its outcome. However, there is currently no supporting evidence for these therapies apart from small studies or case series. Severe cases of COVID-19 may need assisted ventilation through various modalities [4]. A high number of severe cases (especially elderly) may progress to acute severe lung injury and/or multiple organ failure due to a deranged immune response or a “cytokine storm,” the treatment of which may involve immunomodulators. This review covers the key medications suggested as potential agents under investigation in registered clinical trials for the treatment of COVID-19. Broadly, they are either repurposed antiviral, other anti-infective, or immunomodulatory agents, in addition to drugs that act on host cell receptors. The ventilatory and hemodynamic support of critically-ill COVID-19 patients has been reviewed elsewhere [5,6].

The significant advances and continuous progress of computational analysis have greatly served the study of epidemiological aspects COVID-19 through data collection, compilation, and dissemination. In parallel, advances in molecular technology and the availability of the virus genome database have allowed for the identification of the virus and its mutations. An important approach to drug discovery is the screening of chemical libraries, including a large number of available molecules or databases of transcriptional signatures in various cell lines. Ongoing studies are conducted to validate such molecules as potential therapies [7]. 

On the other hand, several products have appeared in the market with strong claims of effectiveness against SARS-CoV-2 infection. Some constitute a risk on patients’ health, e.g., chlorine dioxide products, known as a “Miracle Mineral Solution” that was warned against by the FDA (https://www.fda.gov/news-events/press-announcements/coronavirus-covid-19-update-fda-warns-seller-marketing-dangerous-chlorine-dioxide-products-claim).

The broad strategies for COVID-19 treatment include the targeting of key enzymes of SARS-CoV-2, thus interfering with the viral cycle inside the host cell by using repurposed antiviral drugs previously tested for other coronaviruses like SARS-CoV and MERS-CoV, as well as other RNA virus infections, e.g., HIV and HCV. Other anti-infective agents have also been proposed. In addition, targets on the host cell surface may represent potential COVID-19 therapy-inhibiting virus entries. The second strategy is immunomodulation by using non-specific immunoglobulins and interferons or specific agents, e.g., tyrosine kinase inhibitors and monoclonal antibodies. Immunomodulation is particularly needed early in the disease to boost antiviral immunity, as well as in severe cases where an uncontrolled immune response may lead to acute lung injury and other organ damage.

This review covers key medications proposed for COVID-19 that have been registered in various clinical trials (https://clinicaltrials.gov/), including the Chinese clinical trial database (http://www.chictr.org.cn/index.aspx).

## 2. The SARS-CoV-2 Infection

### 2.1. The Structure of the Virus

**Figure 1:**
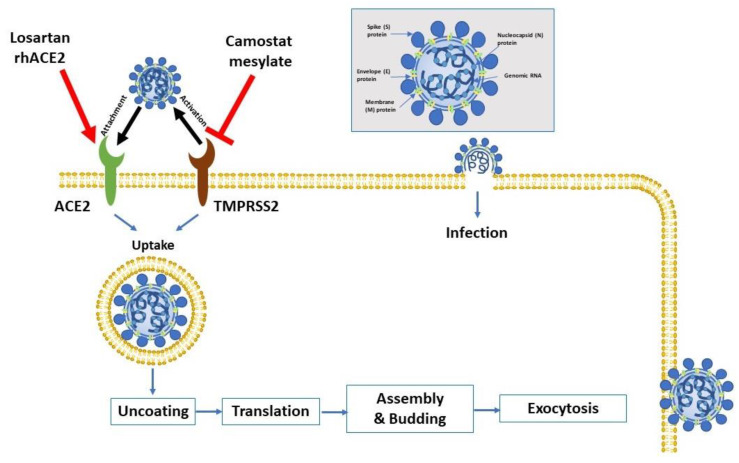
Virus entry into the host cell. The attachment protein “-spike glycoprotein” of the severe acute respiratory syndrome-2 (SARS-CoV-2) uses a cellular attachment factor (angiotensin-converting enzyme 2 (ACE2)) and uses the cellular protease TMPRSS2 (transmembrane protease serine 2) for its activation. ACE2 can be activated via either losartan or recombinant human ACE 2 (rhACE2). Potential pharmacotherapeutic approaches include the use of camostat mesylate (which is a TMPRSS2 inhibitor) to block the priming of the spike protein, increasing the number of ACE2 receptors via losartan, and the use of soluble recombinant human ACE2 (which should slow viral entry into cells via competitive binding with SARS-CoV-2). The structure of SARS-CoV-2 is shown in the upper right.

Coronaviruses belong to Nidovirales order; viruses depending on a nested group of mRNAs for their replication (“nido-” = “nest”). They contain the biggest known viral RNA genomes (27–32 kb in length). These viruses are enveloped positive-sense single-stranded RNA viruses. Figure 1 shows the key virus proteins. Under electron microscopy, the virus has a characteristic crown-like shape due to the presence of the spike (S) protein [8,9]. 

The S protein is extensively glycosylated, forming a homotrimer. It mediates virus entry through binding to specific receptors (e.g., angiotensin-converting enzyme 2 (ACE2)) and fusion with the cell membrane of the host (Figure 1). The S protein harbors the major antigen that stimulates the formation of neutralizing antibodies, in addition to targets of cytotoxic lymphocytes. The membrane (M) protein has a major role in viral assembly [10]. The nucleocapsid (N) protein is responsible for the regulation of viral RNA synthesis—it interacts with M protein during the budding of the virus. It forms part of the nucleocapsid (in association with the RNA). SARS-CoV-2 has sequence homology with the influenza virus through hemagglutinin-esterase glycoprotein (HE). The HE binds to neuraminic acid on the host cell surface, leading to the adsorption of the virus on the cell surface. Such homology may denote an early recombination between the two viruses. Finally, the function of the envelope (E)protein is not fully elucidated. Proteins M, N, and E are essential for the virus’ assembly and release [11].

### 2.2. Clinical Picture

The incubation period is thought to be 14 days after exposure [12]. In more than 80% of patients, the symptoms are mild. However, in 14% of cases, especially in the elderly, the symptoms of dyspnea and hypoxia may develop (with more than 50% lung involvement on computed tomography (CT) scan). In around 5% of cases, in a late phase of the disease, respiratory failure (due to acute lung injury), shock, and/or multiple organ dysfunction (due to the cytokine storm) may develop [13]. Severe cases of COVID-19 that require intensive care unit (ICU) admission have been shown to have increased plasma levels of key inflammatory cytokines including interleukin-2 (IL-2) and tumor necrosis factor-alpha (TNF-α), among many others, thus indicating a cytokine storm that correlates with disease severity [13]. Uncontrolled pulmonary inflammation is a major cause of death in SARS-CoV-2 infection. More than 80% of patients with COVID-19 have lymphopenia [13], suggesting the possible pulmonary recruitment of lymphocytes. Another explanation of lymphopenia is immune-related cell apoptosis or pyroptosis.

## 3. Antiviral Medications

### 3.1. Specific Antiviral Agents

Several approaches have been suggested to target the current SARS-CoV-2 infection in moderate-to-severe cases in an attempt to control viral replication; these approaches include (1) the antiviral repurposing of drugs that were used in SARS-CoV-1 and MERS-CoV, including antiretroviral agents; (2) using immunoglobulins and convalescent plasma; and (3) the bioinformatics screening of chemical libraries for compounds/drugs that are likely to act on SARS-CoV-2. Interestingly, a recent investigation by Klann et al. studied host cell proteomics and highlighted the metabolic pathways particular to the virus, and they came up with medications that are not classified as antiviral—like emetine, which inhibits 40S ribosomal protein S14, and cycloheximide, which is an inhibitor of translation elongation—as potential effective therapies for SARS-CoV-2 infection. The study is under review [14].

#### 3.1.1. Remdesivir (GS-5734)

Remdesivir is an adenosine analog that leads to the premature or delayed termination of the viral RNA chains. The drug was first developed for the treatment of Ebola infection [15,16]. It was found to be a promising antiviral drug against a broad range of viruses (RNA viruses including coronaviruses, e.g., SARS/MERS-CoV5). The drug acts in the post-entry phase, mainly by inhibiting the key enzyme RNA-dependent RNA polymerase (RdRP), [17]. When incorporated into a specific position in the RNA chain, remdesivir causes the inhibition of RNA synthesis at five nucleotides down to the site of the drug incorporation, thus delaying chain-termination [18]. 

Studies in non-human primates have shown the prophylactic and therapeutic value of the drug in the MERS-CoV infection [19]. Another recent in-vitro study reported a half-maximal effective concentration (EC_50_) that suggested the drug is likely to have a plausible working concentration in non-human primates. The investigators concluded that among several drugs tested, only remdesivir (and chloroquine) could inhibit the virus infection in human cells sensitive to SARS-CoV-2 [20].

Recently, on 1 May 2020, the U.S. Food and Drug Administration issued an emergency use authorization of remdesivir for COVID-19 patients. Adverse effects of remdesivir include nausea, vomiting, and liver transaminase elevations. As the used vehicle is cyclodextrin, there is a risk of the potential toxic accumulation of this substance in patients with renal impairment. Remdesivir may be used for pregnant women and children only as compassionate drug use. Remdesivir is under investigation as one of the four arms in the “Solidarity” clinical trial, and its outcome in COVID-19 has been so far described only in case series [21,22]. Several clinical trials are currently investigating the drug; see Table 1.

#### 3.1.2. Lopinavir and Ritonavir

The combination of lopinavir and ritonavir was developed as protease inhibitors for the treatment of HIV-1 infection. The protease enzyme is an aspartic protease that cleaves proteins from precursor viral polypeptide strands. This applies to structural and functional proteins. The proteases play an important role in the viral lifecycle, and, if inhibited, immature, non-infectious virions will be generated [23]. 

Lopinavir undergoes extensive metabolism in the liver by microsomal enzymes “cytochrome 3A4 (CYP3A4)” and “CYP3A5.” Ritonavir inhibits the CYP3A4 enzyme and results in increased concentrations of lopinavir when the two drugs are co-administered [24]. Lopinavir/ritonavir combination is mostly eliminated by the fecal route, whereas the urinary excretion accounts for less than 2% of the eliminated drug. Lopinavir has a low transference across the human placenta; fetal exposure is increased with ritonavir. Based on information collected by the Antiretroviral Pregnancy Registry, the risk of teratogenic effects is not increased in patients receiving lopinavir. Adverse pregnancy outcomes may be linked to the use the antiretroviral therapy during pregnancy, including preterm delivery, stillbirth, low birth weight, and small for gestational age infants [25].

The combination of lopinavir/ritonavir was effective against MERS-CoV in previous studies in experimental animals [23]. Given that success, the combination was recently tried in the treatment of SARS-CoV-2. In contrast, in a study that included 199 adult patients admitted to the hospital with severe COVID-19, lopinavir/ritonavir treatment did not show any benefits compared with the conventional care regarding the time to clinical improvement, viral load, or mortality [26]. However, the combination of lopinavir and ritonavir was included in two arms of the SOLIDARITY trial (with and without the addition of interferon-β). It was also included in another smaller study (150 participants were recruited) in comparison to hydroxychloroquine and a control (no intervention group) for 7–10 days (NCT04307693); see Table 1. Interestingly, a computational study suggested lopinavir (as well as oseltamivir) as a potential therapy for COVID-19 [27].

#### 3.1.3. Darunavir and Cobicistat

Another combination of protease inhibitors was previously reported to be bioequivalent to the lopinavir/ritonavir combination in healthy volunteers, with the advantage of avoiding the adverse effects of ritonavir [28]. Cobicistat is an analog of ritonavir and a potent CYP3A inhibitor. It has no antiviral activity. Like ritonavir, it inhibits CYP2D6 and p-glycoprotein. Cobicistat also inhibits drug transporters, e.g., organic anion transport protein (OATP1B1) which is the main transporter for statins [29]. Clinical trials were registered to compare the combination of darunavir and cobicistat for conventional treatment in patients with COVID-19 (NCT04252274 and ChiCTR2000029541).

#### 3.1.4. Favipiravir

As a guanine analog, favipiravir inhibits the key viral enzyme RNA-dependent RNA polymerase [30]. Favipiravir has been approved for the treatment of influenza viruses type A and B in a few Asian countries. The use of favipiravir in mild cases of COVID-19 was reported by a single study to be associated with a better viral clearance and more frequent radiological improvement compared with lopinavir/ritonavir combined therapy. The analysis was an open-label and non-randomized. Surprisingly, the manuscript was temporarily removed. However, favipiravir has been included in several trials in combination with other antiviral drugs (including lopinavir/ritonavir); see Table 1. 

#### 3.1.5. Ribavirin

The drug is an inosine monophosphate dehydrogenase inhibitor. The enzyme is crucial for the de novo synthesis of guanosine. The drug is mainly used for the treatment of viral hepatitis C. It has not shown a significant effect as a monotherapy in the treatment of SARS-CoV or MERS-CoV [31,32]. However, combined with lopinavir–ritonavir and a corticosteroid, it reduced the 21-day mortality rate due to acute respiratory distress syndrome (ARDS) in patients with SARS-CoV [33]. Ribavirin was also used in combination with lopinavir/ritonavir and an interferon-α (1a or 1b) in the treatment of MERS-CoV and showed no significant effect [34,35]. However, viremia was resolved within two days after starting the combination therapy in severe cases [36]. Interestingly, ribavirin inhibited SARS-COV-2 replication at low concentrations in Caco-2 cells (in micromolar amounts) used as a model for the new viral infection of human cells in a study proposed by Klann et al. (this study is under review). The results of this study are concordant with previous reports of ribavirin effectiveness against other coronaviruses including HCoV-43, CoV-NL63, and MERS-CoV-16, but not SARS-CoV-126. Ribavirin is under investigation in COVID-19 in combination with interferon-α (NCT04254874; ChiCTR2000029308).

#### 3.1.6. Umifenovir

The broad-spectrum antiviral agent, umifenovir, or arbidol hydrochloride, was developed 25 years ago at the Russian Research Chemical and Pharmaceutical Institute. Umifenovir is approved for the prophylaxis and treatment of human influenza type A and type B infections, as well as post-influenza complications (only in a few countries including Russia and China), [37]. Overall, umifenovir has a broad variety of antiviral activity against hepatitis B virus, respiratory syncytial virus, adenovirus, parainfluenza virus, avian coronavirus, coxsackie B3 virus, hantavirus, and arthropod-borne flaviviruses including Zika virus, West Nile virus, and tick-borne encephalitis, thus indicating broad-spectrum antiviral activities [38]. A current trial on mild cases of COVID-19 is investigating the drug as either a single treatment or in combination with pegylated interferon-β atomization for two weeks (NCT04254874).

#### 3.1.7. Oseltamivir

Oseltamivir is a competitive viral neuraminidase enzyme inhibitor. The enzyme inhibition interferes with the release of progeny influenza virus from the infected host cells; thus, new viral cycles are inhibited. After the replication of the virus, progeny virions bind to the infected cell via sialic acid moieties on cell surface glycoproteins. Neuraminidase removes the sialic acid and allows for the release of progeny virions. Thus, neuraminidase inhibitors interfere with the final process of new virion release [39].

Oseltamivir is active against a variety of respiratory viruses, including influenza A and influenza B. Oseltamivir can decrease the severity and duration of the illness if started within 36 h of the onset of symptoms. The influenza neuraminidase enzyme is highly conserved, being common to type A H1N1, type A H2N2, type A H3N2, type A H5N1, and type B influenza viruses [40]. Interestingly, oseltamivir is also active against the strain that caused the 1918 pandemic [41].

Oseltamivir phosphate is available as both a liquid suspension and in a solid formula (capsule or powder). It is a pro-drug that undergoes rapid metabolism to its active form: oseltamivir carboxylate [42]. The drug is eliminated primarily by renal excretion, and dose reduction is recommended for patients with renal impairment.

The most common adverse effects include mild nausea and vomiting (in around 15% of patients). However, post-marketing surveillance has reported rare but serious adverse neuropsychiatric effects (up to convulsions and encephalitis), severe skin reactions (Stevens-Johnson syndrome), and death [43]. Most oseltamivir-related neuropsychiatric events have been reported in children in Japan, where oseltamivir is frequently prescribed [44].

A computational study has suggested oseltamivir as a potential therapy for COVID-19, as it binds to the SARS-CoV-2 protease [27]. A clinical trial was registered to evaluate oseltamivir alone, in combination with ritonavir, or with ASC09F, in treatment of COVID-19. ASC09F is an investigational antiviral interferes with the virus life cycle and will be investigated in combination with oseltamivir and other antiviral agents in at least two trials (NCT04261270 and ChiCTR2000029603). 

### 3.2. Non-Specific Antiviral Agents

#### 3.2.1. Intravenous Immunoglobulins

Intravenous immunoglobulins (IVIG) offer potentially effective therapy in severe cases of COVID-19 through their role in immunomodulation. 

Early in the course of COVID-19 infection, pro-inflammatory cytokines and chemokines are released in response to the rapid rate of virus replication leading to apoptosis [45]. Similar to a previous study on SARS-CoV infection, it can be speculated that viroporin 3a may activate nucleotide-binding and oligomerization domain-like(NOD-like) receptor protein 3 (NLRP3) and inflammasome, thus inducing cell pyroptosis in SARS-CoV-2 infection [46]. Tranilast is an antagonist of NLRP3 that inhibits the inflammasome pathway. Tranilast is a tryptophan analog used to treat allergic conditions, including bronchial asthma, atypical dermatitis, and allergic conjunctivitis [47]. Interestingly, the drug is registered for a Chinese clinical trial in the treatment of COVID-19 (ChiCTR2000030002).

The primary inflammatory responses are usually mild and can be overcome by most patients. It is the secondary inflammatory response that is frequently florid and fatal. Though antiviral neutralizing antibodies enhance the clearance of the virus (as evidenced by the decrease in viral load), anti-spike protein-neutralizing antibodies (anti-S-IgG) in SARS-CoV infection have been found to cause severe lung injury by altering inflammatory responses in previous animal studies [48]. Similarly, the vaccine for SARS-CoV has been found to induce pulmonary injury in multiple animal models [49,50]. 

**Figure 2:**
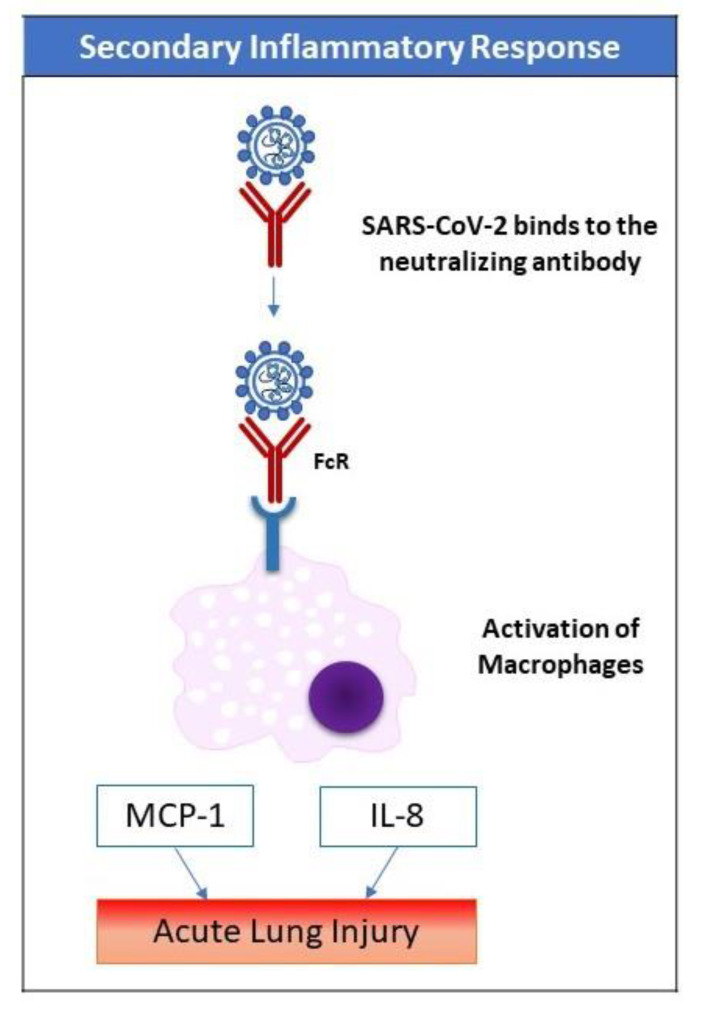
Inflammatory responses triggered by SARS-CoV-2 infection. Two inflammatory pathways may be distinguished. The primary pathway occurs as an early response to viral infection before the development of neutralizing antibodies (NAb). The secondary pathway begins with the release of Nab, which signifies the development of adaptive immunity. Triggering the FcR-mediated-inflammatory response is mediated by the virus-NAb complex and may lead to acute lung injury through several pathways including the release of monocyte chemoattractant protein-1 and interleukin-8 (IL-8) from macrophages.

In COVID-19 patients, the fast development of the anti-S-neutralizing antibody was associated with a higher mortality [51]. The anti-S-IgG may alter the functional polarization of alveolar macrophages and enhance the accumulation of the proinflammatory monocyte/macrophage and the production of monocyte chemoattractant protein-1 (MCP-1) and IL-8 in the lungs. The process starts with the binding of the virus-anti-S-IgG complex to the fragment crystallizable region receptor receptors (FcR) of monocytes/macrophages. Thus, blocking FcR decreases the production of inflammatory cytokines, (Figure 2) [48]. A previously described phenomenon—the antibody-dependent enhancement of viral infection (ADE)—may be a plausible explanation. The process of ADE enhances the cellular uptake of infectious virus-antibody complexes after their interaction with FcR or other receptors, leading to the exacerbation of target cell infection [52].

FcR can be blocked by IVIG, thus suppressing and pacifying the exuberant immune response. Several clinical trials have been registered to investigate the use of intravenous immunoglobulins (NCT04261426 and NCT04264858) or the convalescent plasma of recovered patients (NCT04342195) to treat severe cases of COVID-19. The role of IVIG in severe infections and sepsis is still controversial and warrants further well-designed clinical trials. While a previous randomized trial showed no benefit of IVIG on sepsis-related mortality [2,3,53,54], meta-analyses have shown a reduction in mortality, in particular with the use of IgA- or IgM-enriched IVIG [4,5,55,56].

#### 3.2.2. Interferons

IFN-I is one of the first cytokines released in the context of viral infection. The IFNAR receptors recognize it on the plasma membrane in most cells. Upon interaction with its receptors, interferons induce signal transducer and activator of transcription 1 (STAT1) phosphorylation. STAT1 then translocates to the nucleus to activate interferon-stimulated genes (ISG). The ISGs interfere with viral replication and spread by several mechanisms such as the modulation of cell metabolism or cytokine release, finally activating the adaptive immunity. Pattern recognition receptors (PRRs) are a subgroup of the ISG. PRRs increase the cellular response to pathogens. The ISGs also include proteins that decrease membrane fluidity, thus suppressing the membrane fusion and the egress of the virus. ISGs also comprise antiviral proteins that specifically inhibit steps of the viral cycle [57].

Previously, interferon treatment showed inconclusive activity against SARS-CoV and MERS-CoV. IFNβ1 up-regulates CD73 in the pulmonary endothelium, leading to adenosine secretion. Adenosine is an anti-inflammatory molecule that supports the endothelial barrier. Thus, interferon treatment reduces vascular leakage in ARDS, [58]. Unfortunately, such an anti-inflammatory effect is insufficient to support survival in patients with ARDS [59]. Thus, there is lack of support of use of interferon beta in COVID-19. One assumption of COVID-19-associated severe lung injury is that the virus infection provokes an exaggerated IFN-I-mediated response against the virus, leading to exuberant tissue damage (interferonopathy). Accordingly, IFN-I should be judiciously used by expert specialists. IFN-1 may be administered to patients in the early phase of the disease [60]. However, it is plausible that an anti-interferon agent may be of help in late phases [61].

According to the guidelines implemented in China for COVID-19 infection, IFNα is to be administered by inhalation (five million U twice a day), combined with the antiviral ribavirin [62]. Clinical trials have been recently registered to assess a combination of lopinavir/ritonavir and IFNα2b (ChiCTR2000029387) or a combination of lopinavir/ritonavir with ribavirin and subcutaneously-administered IFNß1b (NCT04276688) for the treatment of COVID-19; see Table 1. 

#### 3.2.3. Thymosin-α1

In addition to its immune-boosting effect, thymosin α1 was recently suggested to facilitate the infiltration of immune cells to improve the effectiveness of checkpoint inhibitors [63]. As an immune-boosting agent, the drug is registered for several clinical trials in combination with antiviral agents for the treatment of COVID-19 (NCT04252274 and ChiCTR2000029541).

## 4. Immuno-Modulatory Agents

### 4.1. Baricitinib

Baricitinib is a small, orally active molecule that inhibits Janus kinase 1 and2. The Janus kinase family, including JAK1, JAK2, JAK3, and tyrosine kinase 2 (Tyk2), has important roles in some signaling pathways through its triggering of the cytokine-induced phosphorylation of STAT that is later transported to the nucleus to regulate gene transcription [64,65].

Baricitinib is reported to be effective in the treatment of rheumatoid arthritis in several clinical trials [65], with excellent results in terms of clinical response and a good safety profile [66]. The literature has reported that the drug blocks the phosphorylation of STATs induced by different cytokines in the blood, and it causes a transient alteration in neutrophil and lymphocyte counts [66,67,68,69].

Receptor-mediated endocytosis is the most common pathway for virus entry to the cells. ACE2, a cell surface protein expressed by cells in the kidney, blood vessels, heart, and, most importantly, lung AT2 alveolar epithelial cells, is the receptor used by SARS-CoV-2 to infect lung cells [70]. AP2-associated protein kinase 1 (AAK1) is a pivotal regulator of clathrin-mediated endocytosis; thus, the disruption of AAK1 would interfere with the virus passage into the cells and further prevent the intracellular assembly of virus particles [71]. 

A benevolent Artificial intelligence (AI)s knowledge graph and customizations bespoke to SARS-CoV-2 were used together to identify drugs that may halt the viral infection process. Baricitinib was expected to have a high binding affinity to AAK1 and cyclin G-associated kinase, another regulator of endocytosis [72]. Accordingly, baricitinib is suggested to be trialed on patients with SARS-CoV-2 acute respiratory disease in order to reduce the viral entry and the associated inflammation (NCT04321993).

A great chance for potential combination therapy with baracitinib is present due to its favorable pharmacokinetic properties such as low plasma protein binding affinity, minimal interaction with CYP enzymes, and drug transporters [73]. Currently, in the COVID-19 outbreak, baricitinib is being combined with different antivirals, such as lopinavir, ritonavir, and remdesivir, since this combination would decrease viral infectivity, viral replication, and the exaggerated host inflammatory response.

On the other hand, using baricitinib is associated with an increased risk of developing severe infections due to opportunistic pathogens, especially if it is taken concomitantly with immunosuppressants such as corticosteroids [74]. Tuberculosis was identified in patients receiving baricitinib, and patients should be tested prior to and during therapy for latent tuberculosis infection. In addition, thrombosis, including deep venous thrombosis and pulmonary embolism, has been documented more than placebo in patients treated with baricitinib [74].

### 4.2. Ruxolitinib

Ruxolitinib is an orally available and potent JAK1 and JAK2 inhibitor. It was the first drug for myelofibrosis to be approved by the FDA in November 2011 [75]. The pharmacokinetic analysis of ruxolitinib predicted that the unbound plasma concentration required to inhibit clathrin-mediated endocytosis greatly exceeds the currently tolerated therapeutic dose [73]. Therefore, viral infectivity at therapeutic doses is unlikely to decrease, but the drug may reduce the inflammatory response of the host by inhibiting JAK. Ruxolitinib is currently being studied to combine it with COVID-19 mesenchymal stem cell infusion (NCT04252118).

### 4.3. Camrelizumab

Camrelizumab is a humanized monoclonal antibody against programmed cell death 1 (PD-1) receptors. PD-1 receptors are expressed on the surface of T cells and act as negative regulators of T cell function. Camrelizumab has been reported to block PD-1′s binding to its ligand, PD-L1, thus inhibiting the immune escape of tumor cells [76]. Recently, in China, the drug has received conditional approval for the treatment of relapsed or refractory Hodgkin’s lymphoma. Camrelizumab is also being investigated as a possible therapy for other malignancies [76].

After antigen exposure, the expression of PD-1 on T-cells is induced rapidly, following the engagement between the T-cell receptor (TCR) and the loaded major histocompatibility complex (MHC) molecule in the draining lymph nodes [77]. The presence of various pro-inflammatory mediators such as IL-2, IL-6, IL-7, and IL-15 in the affected tissue contribute further to PD-1′s up-regulation of T-cells [78]. The interaction between PD-1 and PD-1L provides signals that tolerize T-cells to their antigenic targets, neutralizing their effector functions [78]. Furthermore, in sepsis patients, PD-1 and PD-L1 are known to be the primary mediators in T cell depletion [79]. Animal models have shown that blocking PD-1 or PD-L1 prevents T cell death, regulates cytokine production, and reduces both organ dysfunction and death in sepsis [80]. Researchers are therefore attempting to perform clinical trials to determine the effectiveness of PD-1 inhibitors in 2019 novel coronavirus infection in patients with serious lymphocytopenia-associated pneumonia.

On the other hand, the use of camrelizumab is associated with a good safety profile; the high incidence of all adverse drug reactions is related to reactive capillary hemangiomas, which are generally unthreatening and self-limited [81]. Currently, camrelizumab is being investigated for its effectiveness in COVID-19 (NCT04268537).

### 4.4. Eculizumab

Eculizumab is a humanized monoclonal IgG antibody that binds to the C5 complement protein, preventing cleavage into C5a and C5b. Blocking the formation of C5b prevents terminal complex C5b-9 or membrane attack complex (MAC) from subsequent formation. In the United States, eculizumab is approved for treating paroxysmal nocturnal hemoglobinuria and atypical hemolytic uremic syndrome [82,83].

The literature has identified the complement system as an essential host mediator for both SARS-CoV- and MERS-CoV-induced disease and has suggested that complement activation controls the systemic pro-inflammatory response against the viral infection [84,85]. Accordingly, eculizumab is expected to stop immune-mediated death in patients infected with SARS-CoV-2. Currently, the drug is under clinical trials for its effectiveness in patients with confirmed COVID-19 infection who are in ICU due to ARDS (NCT04288713). 

### 4.5. Meplazumab 

Meplazumab is a humanized anti-CD147 antibody. A recent study showed that SARS-CoV-2 can invade human host cells via a novel route of the CD147-spike protein (SP), [86]. The SP was found to bind to CD147, a receptor on the host cells, thus mediating the invasion of the virus. The study also showed that meplazumab could competitively inhibit the binding of SP and CD147, and it could prevent viruses from invading host cells; this has provided a potential target for developing antiviral cures or protocols.

Recently, the drug was evaluated for its effectiveness and safety as an add-on treatment in patients with COVID-19 pneumonia. Meplazumab was found to speed up the recovery of patients with pneumonia associated with COVID-19 infection with a favorable safety profile [87]. The findings of this study support the carrying out of a large-scale investigation of meplazumab as a possible treatment for COVID-19 pneumonia.

### 4.6. Tocilizumab

Tocilizumab is a humanized monoclonal antibody against the interleukin-6 receptor (IL-6R). IL-6 is a cytokine that plays a major role in immune response and is associated with the pathogenesis of many autoimmune diseases [88]. IL-6 is produced by different cells, including T-cells and B-cells, lymphocytes, monocytes, and fibroblasts. IL-6 is involved in various physiological processes such as T-cell activation, the induction of immunoglobulin secretion, the initiation of hepatic acute-phase protein synthesis, and the stimulation of hematopoietic cells precursor proliferation and differentiation. The drug is already approved for the treatment of serious or life-threatening cytokine release syndrome (CRS). The FDA has approved a randomized, double-blind, placebo-controlled phase III clinical trial to assess the safety and efficacy of combining tocilizumab to the standard of care in hospitalized patients with severe COVID-19 pneumonia [89].

The seventh edition of the Chinese Clinical Guidance for COVID-19 Pneumonia Diagnosis and Treatment published by China National Health Commission on 4 March 2020 included tocilizumab as an option for patients with severe COVID-19, extensive lung lesions, and elevated IL-6 levels [90]. This followed reports of positive outcomes from the use of tocilizumab to control dangerous lung inflammation in 21 patients with severe COVID-19 in China [91].

However, Health Canada notified healthcare providers that severe cases of drug-induced liver injuries had been reported in patients treated with tocilizumab, including cases of acute liver failure that required a transplant [92]. Patients should be closely monitored for the emergence of signs and symptoms of infection during and after treatment with tocilizumab, including the potential development of tuberculosis in patients who have tested negative for latent tuberculosis infection before starting therapy. Many patients who acquired these infections were taking concomitant immunosuppressants, such as methotrexate or corticosteroids, and, as such, the drug should be only used according to the specialist instructions [93].

### 4.7. Sarilumab

Sarilumab is another human monoclonal antibody against the interleukin-6 receptor. It binds to soluble and membrane-bound IL-6 receptors and inhibits IL-6-mediated signaling via these receptors. There is a growing body of evidence confirming the benefit of targeting the IL-6 pathway in patients with COVID-19, most notably in a recent single-arm study in China with tocilizumab in critically ill COVID-19 patients [94]. 

Currently, the clinical efficacy of sarilumab against SARS-CoV-2 is being evaluated in various clinical trials (Table 1).

Similar to tocilizumab, sarilumab-treated patients are at an elevated risk of developing severe opportunistic infections that may result in hospitalization or death [95].

### 4.8. Bevacizumab

Bevacizumab is a recombinant, humanized monoclonal antibody that has been used in anti-tumor therapy for 16 years. It binds to and neutralizes vascular endothelial growth factor (VEGF), preventing its interaction with endothelial receptors, Flt-1 and KDR. The suppression of vascular permeability in patients with severe/critical COVID-19 is expected to reduce pulmonary edema. The autopsies of patients who died from COVID-19 have shown the significant exudation of the pulmonary mucus, more evident than infection with SARS [96]. Pulmonary CT scanning and pathological observation have recognized inflammatory exudation as a distinctive characteristic of COVID-19 [97]. Evidence has suggested that bevacizumab is a promising treatment for critically ill COVID-19 patients. Currently, the drug is being evaluated for its efficacy and safety in severe or critical patients with COVID-19 in a multicenter, randomized, controlled clinical trial [98].

Bevacizumab has been reported by the American Heart Association to be an agent that may either cause reversible direct myocardial toxicity or exacerbate underlying myocardial dysfunction Moreover, bevacizumab has been reported to cause and/or worsen hypertension [98]. Thus, blood pressure monitoring during bevacizumab treatment and regularly after discontinuation is recommended.

### 4.9. Fingolimod

Fingolimod is a sphingosine analog that modulates the sphingosine-1-phosphate receptor and thus alters the migration of lymphocytes, resulting in lymph node sequestration [99]. As an immunomodulatory agent, it may confine the over-exuberant inflammatory response and slow down the progress of lung injury.

Fingolimod is mainly contra-indicated in patients with myocardial infarction (within six months), unstable angina, stroke, heart failure, and heart block. Prolonged QT interval above 500 m/s is another important contra-indication, given the high incidence of myocarditis as a complication of severe COVID-19. Patients with diabetes may get macular edema upon fingolimod treatment [100]. A clinical trial has been registered to use fingolimod at a dose of 0.5 mg daily for three consecutive days in the treatment of severe COVID-19 infection (NCT04280588).

## 5. Other Anti-Infective Agents Repurposed to Treat COVID-19

### 5.1. Chloroquine and Hydroxychloroquine

Both chloroquine (CQ) and hydroxychloroquine (HCQ) are antimalarial drugs that are also prescribed in treating autoimmune diseases [101]. According to the literature, both chloroquine and hydroxychloroquine possess antiviral activity against several viruses. CQ, through the inhibition of specific viral replication steps, restricts HIV. [102], influenza virus [103], dengue virus (DENV), [104], West Nile virus (WNV) infection, Japanese encephalitis virus (JEV), and Zika virus infection [105]. Those specific viral replication steps depend on the pH.

The investigation of the anti-SARS-CoV-2 activity of CQ and HCQ is also encouraged by several factors. First, both CQ and HCQ have been used for the treatment and prophylaxis of malaria and rheumatic diseases for more than 50 years. Second, both drugs are very cheap, e.g., when compared with immunotherapy. Finally, CQ inhibits the synthesis of a myriad of pro-inflammatory cytokines such as TNF-α, IL-1, and IL-6 [106], which may have deleterious effects on COVID-19 prognosis.

CQ and HCQ, as weak bases, exert their antiviral mechanism by affecting the acid vesicles, which leads to the disruption of enzymes required for the post-translational modifications of proteins. Both drugs concentrate in the acidic cell organelles, thus inhibiting virus replication by preventing endosome/lysosome trafficking. Another potential mechanism is the intervention with the viral protein maturation during virion maturation [107]. CQ has been shown to alkalinize the endosomal pH, thus preventing fusion between the virus and the cell and interfering with the glycosylation of the ACE2 receptor and its binding to the SARS-CoV-2 spike protein [108]. 

To date, clinical studies investigating hydroxychloroquine or chloroquine have been limited, and their role in COVID-19 treatment is still to be elucidated. Recent reports have found that CQ can also inhibit the growth of SARS-CoV-2 [20,108]. Chinese experts recommend that all cases of COVID-19 who have no contraindications to CQ should receive the drug in a dose of 500 milligrams twice daily for ten days [109]. 

In the United States, the FDA endorsed an emergency use authorization to permit CQ and HCQ treatment for hospitalized patients for COVID-19 [109]. There is a risk of potential toxicity with those medications, including QTc prolongation, cardiomyopathy, and retinal toxicity. Given that, and because of the previously described cases of myocarditis in COVID-19, CQ and HCQ should be cautiously prescribed under close clinical supervision by specialists. Patients with myocarditis are expected to be more susceptible to cardiac adverse effects. In addition, drug–drug interactions should be considered before use, and the patients should be monitored closely for adverse effects during use. The American College of Cardiology has recommended QTc monitoring parameters in that setting [110]. 

In a study on 36 patients with COVID-19, patients on HCQ (200 mg three times per day for 10 days) showed a higher rate of undetectable SARS-CoV-2 RNA on nasopharyngeal specimens at day six compared to patients who received no specific treatment (70% versus 12.5%, respectively) [111]. In this study, the use of azithromycin in combination with HCQ was apparently associated with a more rapid decline in viral RNA; however, the control groups for the study were a concern, and the biologic basis for using azithromycin in this setting is unclear. Another small observational study in patients with more severe illness did not suggest rapid viral RNA clearance with the combination [112].

### 5.2. Ivermectin

Ivermectin is derived from avermectins produced by the microorganism *Streptomyces avermitilis*, which was discovered by Ōmura [113]. Ivermectin, which is a highly safe compound, was later marketed to cover agricultural and aquaculture as well as the veterinary areas in 1981. Ivermectin, in the oral tablet dosage form, is used to treat infections of parasites, including the intestinal tract, skin, and eyes parasitic infections.

Ivermectin has numerous medical uses in which antiviral activity is considered one of the experimental targets. Ivermectin in sub-nanomolar range EC_50_ inhibits the replication of the yellow fever virus [113]. Ivermectin is also able to inhibit other flavivirus replication, including: dengue, tick-borne encephalitis, and Japanese encephalitis viruses. One of the mechanisms involved in this inhibition is the targeting of the activity of the non-structural enzyme 3 helicase [113]. Moreover, ivermectin inhibits dengue viruses and interrupts virus replication [114]. Ivermectin has also been demonstrated to be a potent inhibitor of importin α/β, which mediates the nuclear transport of several RNA viruses. Thus, ivermectin blocks the nuclear trafficking of RNA viral proteins. Ivermectin has been shown to have potent antiviral activity against HIV-1 and dengue viruses. Both types of viruses depend on the importin protein system. Ivermectin may also disrupt the HIV-1 integrase in HIV-1 in addition to the NS-5 (non-structural protein 5) polymerase in dengue viruses [115,116]. 

To investigate the effect of ivermectin on SARS-CoV-2, Vero/hSLAM cells with SARS-CoV-2 that were isolated Australia/VIC01/2020 were treated with ivermectin [117]. Ivermectin administration resulted in a successful viral loss by 48 h with no further reduction in viral RNA at 72 h. The authors hypothesized that ivermectin exerts its antiviral activity by inhibiting the nuclear import of viral proteins that is mediated by importinα/β1—the same mechanism discussed before for other RNA viruses.

### 5.3. Azithromycin

Macrolides are a unique class of antibiotics that include erythromycin, clarithromycin, and azithromycin. They have not only antimicrobial activity but also immunomodulatory reactions, including anti-inflammatory effects. Lately, the anti-viral effects of macrolides have been investigated.

Erythromycin is the first macrolide proved to have efficacy in the treatment of rhinovirus and influenza virus [118]. On the other hand, clarithromycin and azithromycin have shown higher effectivity towards rhinovirus, respiratory syncytial virus, and influenza virus [118,119]. In addition, Zika and Ebola viruses have been shown to be inhibited by azithromycin [120,121]. During the early phase of influenza virus infection, azithromycin has been found to block influenza virus endocytosis into host cells [119].

Regarding macrolide treatment for COVID-19, Gautret et al. hypothesized that hydroxychloroquine, in addition to azithromycin, might be an effective treatment for COVID-19. The mechanism of azithromycin against SARS-CoV-2 is unclear; however, it may be similar to the mechanistic pathways of macrolides against the influenza virus [111].

Several pathways for the suspected antiviral effects found with azithromycin have been suggested. Firstly, as mentioned regarding CQ and HCQ, azithromycin is a weak base that could inhibit endocytosis, thus limiting viral entry and replication [122]. Secondly, azithromycin directly affects the activity of bronchial epithelial cells and decreases mucus production, thus improving lung function [123]. Thirdly, there is evidence suggesting that azithromycin increases the production of IFN via the stimulation of IFN genes, leading to a reduction of viral replication [124]. Lastly and recently, azithromycin was shown to directly intervene with the SARS-CoV-2 host cell entry by inhibiting the interaction between SARS-CoV-2 spike protein and the host cell receptor, ACE2 [125].

## 6. Drugs Acting on Host Cell Receptors

### 6.1. Angiotensin-Converting Enzyme Inhibitors (ACEis) and Angiotensin II Receptor Blockers (ARBs)

Similar to SARS-CoV, SARS-CoV-2 binds first with its S glycoprotein to angiotensin-converting enzyme 2 (ACE-2), a homologs enzyme to ACE expresses on the membrane of lung epithelial cells, which is followed by virus-cell fusion and viral entry; see Figure 1, [125,126,127,128]. ACE-2 is a zinc carboxypeptidase whose catalytic active site is exposed at the extracellular surface [126,129]. Angiotensin II (ANGII) is generated from angiotensin I (ANGI) after being cleaved by ACE. ANGII binds to angiotensin type 1 receptor (AT1R) and functionally results in blood pressure elevation. ACE2 terminates the ANGII effect by degrading it into angiotensin-(1–7), which binds to Mas receptors on blood vessels, thus resulting in a vasodilator effect [12]. Furthermore, ACE2 degrades ANGI into angiotensin-(1–9), which is later converted into angiotensin-(1–7) via ACE [130]. Notably, ACE2 acts as a negative regulator in the renin–aldosterone–angiotensin system and opposes the deleterious cardiovascular effects induced by that system [126,129]. ANGII plays an important role in regulating ACE2. In the absence of abnormally elevated levels of Ang II, complex formation between angiotensin receptor (AT1R) and ACE2 on the cell membrane is predominant [129]. When ANGII levels increase, the AT1R/ACE2 complex is disrupted and triggers ACE2 cellular internalization, with subsequent lysosomal degradation via an AT1R-dependent pathway [129,131].

Angiotensin-converting enzyme inhibitor (ACEi) and angiotensin II receptor blocker (ARB) are two related categories of antihypertensive treatment that are extensively used to treat hypertension and other cardiovascular diseases. ACEi and ARB increase the expression of ACE2 on the lung epithelial cell surface [126,129]. Therefore, it has been suggested that treatment with ACEi or ARB could have a higher expression of cell membrane-bound ACE2, exposing essential binding sites to SARS-CoV-2 spike glycoprotein. However, the reduction of ANGII synthesis by ACEi or the inhibition of ANGII’s physiological effect via ARB is thought to leave AT1R in interaction with ACE2 [129]. This interaction could block the affinity of SARS-CoV-2 spike glycoprotein to ACE2 and hence reduce SARS-CoV-2 viral entry [129]. Furthermore, Dijkman et al. suggested that viral replication directly diminishes cellular ACE2 levels [130]. This may explain the lung protection role for ACEi and ARB via increasing the ACE2 levels and opposing the SARS-CoV-2 infection consequences.

Losartan is an AT1R blocker that is widely applied in the management of hypertension and to slow down the nephropathy progression in patients with diabetes mellitus. It is a safe drug with infrequent adverse drug events [132]. Interestingly, around 50% of hospitalized SARS-CoV patients have developed hypotension during their hospital stay [133]. No published clinical data are yet available on hypotension rates among hospitalized (NCT04312009) or non-hospitalized (NCT04311177). SARS-CoV-2 patients; it is thus too early to estimate how safe AT1R blockers in targeting COVID-19 without developing exacerbated hypotension.

Another approach for treatment of COVID-19 is to use a soluble form of ACE2. A recombinant human ACE2 (rhACE2; APN01, and GSK2586881) is safe in patients with acute respiratory distress syndrome [134,135]. Treatment with rhACE2 rapidly abolishes the elevated levels angiotensin-II, with a trend to decrease the plasma concentration of IL-6. A small clinical trial of rhACE2 in patients with severe SARS-CoV-2 infection was recently initiated (NCT04287686).

Logically, low cellular membrane ACE2 expression may reduce the chance of cells to be entered by SARS-CoV-2, but at the same time, it leads to the activation of AT1R with subsequent lung tissue injury. However, the higher the expression of ACE2 on the lung cell surface, the higher the chance of viral entry and the lower chance lung injury, due to minimal AT1R activation. This is the main reason why ACEi/ARB or rhACE2 may be useful in COVD-19 treatment. These opposing effects of ACE2 and, hence, ACEi/ARB or rhACE2, treatment in SARS-CoV-2 infection need more investigation.

### 6.2. Camostat

In-vitro studies have shown that endosomal cysteine proteases, namely cathepsin B (CTSB) and L (CTSL), can activate the glycoproteins of filoviruses, SARS-CoV, other coronaviruses, Nipah virus (NiV), and Hendra virus (HeV) through a mechanism that facilitates virus entry into certain cell lines [136]. In addition, other molecules that can lead to the activation of coronaviruses include TMPRSS2, and other serine proteases present extracellularly or present at the cell surface [137]. TMPRSS2 activity is crucial for viral spread and pathogenesis in an infected host [16,138], and camostat mesylate is a serine protease inhibitor that inhibits SARS-CoV activation by TMPRSS [139]. Camostat mesylate also inhibits the replication of both influenza and parainfluenza viruses. In experimentally infected mice, camostat was also found to prevent the development of pneumonia and viral myocarditis [140]. Moreover, camostat mesylate has been found to help to slow down or inhibit chronic pancreatitis in animal models and has been used for the treatment of chronic pancreatitis patients in Japan [141]. Therefore, camostat mesylate could also be of benefit in patients infected with SARS-CoV-2 (Table 1). 

## 7. Conclusions

The drugs presented in this review are possible pharmacological modalities to COVID 19, but most of them are proposed based on their possible pharmacological mechanism to target virus entry, replication, and associated complications. The management of COVID 19 is rapidly evolving, as all available therapies are currently being investigated in clinical trials, and information will continue to emerge regarding pharmacological therapy for SARS-CoV-2. While awaiting the further development of clinical guidelines, the rational use of therapies should be ensured by local protocols based on the clinical expertise.

## Figures and Tables

**Table 1:** Pharmacology of coronavirus disease (COVID-19) therapies under clinical trials.

Drug	Mechanism of Action	Route of Administration	Adverse Effects	Contra-Indications	Drug–Drug Interactions	Registered Clinical Trials in COVID-19
**Specific Antiviral Agents**						
Remdesivir	Inhibits viral RdRp	Intravenous	Elevated ALT/AST (reversible); nephrotoxicity	Known hypersensitivity	Non-significant	https://clinicaltrials.gov/ct2/show/NCT04330690 https://clinicaltrials.gov/ct2/show/NCT04365725 https://clinicaltrials.gov/ct2/show/NCT04292899 https://clinicaltrials.gov/ct2/show/NCT04292730 https://clinicaltrials.gov/ct2/show/NCT04252664 https://clinicaltrials.gov/ct2/show/NCT04257656 https://clinicaltrials.gov/ct2/show/NCT04280705
Lopinavir/Ritonavir	Inhibits viral 3-chymotrypsin-like protease	Oral	Gastrointestinal, nausea, vomiting, and diarrhea. Pancreatitis, hepatotoxicity, and cardiac conduction abnormalities	Known hypersensitivity, co-administration with drugs highly dependent on CYP4503A, and co-administration with potent CYP450 3A inducers	Ritonavir: CYP3A4 inhibitor and substrate; CYP2D6 substrate; CYP1A2, CYP2B6, CYP2C8, CYP2C9, CYP2C19 inducer. P-glycoprotein substrate; and 1 inducer	https://clinicaltrials.gov/ct2/show/NCT04376814 https://clinicaltrials.gov/ct2/show/NCT04321174 https://clinicaltrials.gov/ct2/show/NCT04307693 https://clinicaltrials.gov/ct2/show/NCT04350684 https://clinicaltrials.gov/ct2/show/NCT04346147 https://clinicaltrials.gov/ct2/show/NCT04261907 https://clinicaltrials.gov/ct2/show/NCT04276688 https://clinicaltrials.gov/ct2/show/NCT04330690
Darunavir/Cobicistat	Inhibits viral 3-chymotrypsin-like protease	Oral	Dizziness, sleep disturbances, and altered sensorium. GIT upset, headache, skin rash, asthenia, and redistribution of fat	Known hypersensitivity	Cobistat: CYP3A and 2D6 inhibitor; p-glycoprotein and OATP1B1 inhibitor	https://clinicaltrials.gov/ct2/show/NCT04252274 http://www.chictr.org.cn/showprojen.aspx?proj=48992
Favipiravir	Inhibits viral RdRp	Oral	Anemia, neutropenia, hyperuricemia, diarrhea, and elevated ALT/AST.	Known hypersensitivity	CYP2C8 and aldehyde oxidase inhibitor	https://clinicaltrials.gov/ct2/show/NCT04376814
Ribavirin	Inhibits viral RdRp	Oral	Flu-like symptoms, depression, suicide, insomnia, irritability, relapse of drug abuse/overdose, and anemia	Known hypersensitivity	Non-significant	https://clinicaltrials.gov/ct2/show/NCT04276688
Umifenovir (arbidol)	Inhibits spike protein/ACE2 interaction	Oral	Hypersensitivity	Allergic reaction, GIT upset, and elevated ALT/AST	Non-significant	https://clinicaltrials.gov/ct2/show/NCT04350684
Oseltamivir	Neuroaminidase inhibitor	Oral	Nausea, vomiting, psychiatric effects, and nephrotoxicity	Known hypersensitivity	Non-significant	https://clinicaltrials.gov/ct2/show/NCT04261270 https://clinicaltrials.gov/ct2/show/NCT04303299 http://www.chictr.org.cn/searchprojen.aspx?regno=ChiCTR2000029603&title=&officialname=&subjectid=&secondaryid=&applier=&studyleader=&ethicalcommitteesanction=&sponsor=&studyailment=&studyailmentcode=&studytype=0&studystage=0&studydesign=0&minstudyexecutetime=&maxstudyexecutetime=&recruitmentstatus=0&gender=0&agreetosign=&secsponsor=&regstatus=0&country=&province=&city=&institution=&institutionlevel=&measure=&intercode=&sourceofspends=&createyear=0&isuploadrf=&whetherpublic=&btngo=btn&verifycode=&page=1
**Non-Specific Antiviral Agents**						
Intravenous Immunoglobulins	Boosts the antiviral immune response	Intravenous	Mild, transient, reversible events such as headaches, chills, or flushing; an increased risk of thrombosis, renal dysfunction, and acute renal failure	IgA deficiency and prior hypersensitivity reactions are not contraindications.	Non-significant	https://clinicaltrials.gov/ct2/show/NCT04350580 https://clinicaltrials.gov/ct2/show/NCT04348877 (antibody-rich plasma)
Interferons	Boosts the antiviral immune response in the early phase of the disease	subcutaneous	GIT upset, urinary urgency, leukopenia, lymphocytopenia, neutropenia, and elevated ALT/AST Inflammation at injection site, ataxia, headache, insomnia, asthenia, and flu-like symptoms	Hypersensitivity, pregnancy, current severe depression or suicidal ideation, and liver failure.	Inhibits metabolism of zidovudine	https://clinicaltrials.gov/ct2/show/NCT04350684 (IFNβ-1) https://clinicaltrials.gov/ct2/show/NCT04276688 (IFNβ-1) https://clinicaltrials.gov/ct2/show/NCT04350671 (IFNβ-1) https://clinicaltrials.gov/ct2/show/NCT04343976 (IFN-λ)
**Immuno-Modulatory Agents**						
Baricitinib	JAK-1 and JAK-2 inhibitor	Oral	Serious infections, malignancies, thrombosis, increased serum, alanine, aminotransferase, and serum aspartate aminotransferase	History of venous thromboembolism active or latent tuberculosis infection, pregnancy and lactation serious acute infections, solid-organ transplant recipient, ALT/AST > 5 × upper limit of normal, absolute neutrophil count < 1 × 10^9^ cells/L, absolute lymphocyte count < 0.2 × 10^9^ cells/L, hemoglobin < 8 g/dL, estimated glomerular, and filtration rate (GFR) < 30 mL/min/1.73 m^2^	It is a substrate of BCRP/ABCG2, CYP3A4 (minor), OAT1/3, and P-glycoprotein/ABCB1. Potentially significant interactions may exist	https://clinicaltrials.gov/ct2/show/NCT04358614 https://clinicaltrials.gov/ct2/show/NCT04340232 https://clinicaltrials.gov/ct2/show/NCT04346147 https://clinicaltrials.gov/ct2/show/NCT04320277 https://clinicaltrials.gov/ct2/show/NCT04373044 https://clinicaltrials.gov/ct2/show/NCT04321993 https://clinicaltrials.gov/ct2/show/NCT04345289
Ruxolitinib	JAK1 and JAK2 inhibitor	Oral		Uncontrolled HIV infection, active tuberculosis, chronic kidney disease requiring dialysis, ALT/AST > 5 times the upper limit of normal, and pregnancy or lactation. Known or expected to have allergic reactions to the drug	Substrate for CYP3A4 Potentially significant interactions may exist, requiring dose or frequency adjustment	https://clinicaltrials.gov/ct2/show/NCT04348071 https://clinicaltrials.gov/ct2/show/NCT04359290 https://clinicaltrials.gov/ct2/show/NCT04355793 https://clinicaltrials.gov/ct2/show/NCT04354714 https://clinicaltrials.gov/ct2/show/NCT04362137 https://clinicaltrials.gov/ct2/show/NCT04377620 https://clinicaltrials.gov/ct2/show/NCT04366232 https://clinicaltrials.gov/ct2/show/NCT04334044 https://clinicaltrials.gov/ct2/show/NCT04374149 https://clinicaltrials.gov/ct2/show/NCT04338958 https://clinicaltrials.gov/ct2/show/NCT04337359 https://clinicaltrials.gov/ct2/show/NCT04331665 https://clinicaltrials.gov/ct2/show/NCT04361903 https://clinicaltrials.gov/ct2/show/NCT04348695
Camrelizumab	Programmed cell death 1 (PD-1) blocking antibody	Intravenous	Reactive skin capillary hyperplasia, hypothyroidism pneumonia, asthenia, leukopenia, and neutropenia	Pregnancy or lactation; known or expected to have allergic reactions to the drug; autoimmune diseases; history of organ, bone marrow, or hematopoietic stem cell transplantation; and received radiotherapy and chemotherapy for malignant tumor within six months	N/A	https://clinicaltrials.gov/ct2/show/NCT04268537
Eculizumab	Complement Inhibitor	Intravenous	Increases the risk of meningococcal infections, paroxysmal nocturnal hemoglobinuria hemolytic uremic syndrome, and generalized asthenia	Pregnancy or lactation, history or unresolved, Neisseria meningitis infection, ongoing sepsis, and the presence or suspicion of active and untreated systemic bacterial infection allergy	Minor drug interactions may exist	https://clinicaltrials.gov/ct2/show/NCT04346797 https://clinicaltrials.gov/ct2/show/NCT04355494 https://clinicaltrials.gov/ct2/show/NCT04288713
Meplazumab	Anti-CD147 antibody	intravenous	No adverse effects were reported in meplazumab-treated patients.	Known or expected to have allergic reactions to the drug	N/A	https://clinicaltrials.gov/ct2/show/NCT04275245
Tocilizumab	Interleukin-6 Receptor Antagonist	Intravenous	Patients treated with tocilizumab are at an increased risk for developing serious infections that may lead to hospitalization or death. Most patients who developed these infections were taking concomitant immunosuppressants, such as methotrexate or corticosteroids.	Known or expected to have allergic reactions to the drug	It may enhance the immunosuppressive effect of biologic disease-modifying antirheumatic drugs (DMARDs).	https://clinicaltrials.gov/ct2/show/NCT04275245
Sarilumab	Interleukin-6 Receptor Antagonist	Subcutaneous	Elevated ALT/AST	Known or expected to have allergic reactions to the drug	It may enhance the immunosuppressive effect of DMARDs.	https://clinicaltrials.gov/ct2/show/NCT04359901 https://clinicaltrials.gov/ct2/show/NCT04357808 https://clinicaltrials.gov/ct2/show/NCT04315298 https://clinicaltrials.gov/ct2/show/NCT04357860 https://clinicaltrials.gov/ct2/show/NCT04327388 https://clinicaltrials.gov/ct2/show/NCT04324073 https://clinicaltrials.gov/ct2/show/NCT04345289 https://clinicaltrials.gov/ct2/show/NCT04322773 https://clinicaltrials.gov/ct2/show/NCT02735707
Bevacizumab	Antibody against the vascular endothelial growth factor (VEGF)	Intravenous	Some studies only reported hematologic toxicities grades ≥4 and nonhematologic toxicities grades ≥3.	Known or expected to have allergic reactions to the drug	It may enhance the cardiotoxic effect of anthracyclines and the myelosuppressive effect of myelosuppressive agent	https://clinicaltrials.gov/ct2/show/NCT04344782 https://clinicaltrials.gov/ct2/show/NCT04305106 https://clinicaltrials.gov/ct2/show/NCT04275414
Fingolimod	Sphingosine 1-phosphate receptor modulator	Oral	headache, QTc prolongation asthenia, stuffy nose, sinus pain, diarrhea, and elevated AST/ALT	A baseline QTc interval ≥ 500 msec, heart block, CAD, pregnancy, and known hypersensitivity	Ketoconazole increases the drug level; vaccination may be less effective	https://clinicaltrials.gov/ct2/show/NCT04280588
**Other Anti-Infective Agents Repurposed to Treat COVID-19**						
Chloroquine and hydroxychloroquine	Inhibits viral entry and endocytosis	Oral	QTc prolongation, hypoglycemia, neuropsychiatric effects, and retinopathy	Asian patients Ocular disease Visual disturbance Porphyria Psoriasis Alcoholism Hepatic disease GIT disease G6PD deficiency Myopathy Neurological disease Hypoglycemia AV block Bradycardia Cardiomyopathy Celiac disease Heart failure HIV infection Hyperparathyroidism Hypocalcemia Hypokalemia Hypomagnesemia Hypothyroidism Long QT syndrome	Arsenic trioxide Methotrexate Acetaminophen Iron products Kaolin Niacin Rifampin Isoniazid Antiarrhythmic Anti-depressants Vitamins and herbal products Antacids Insulin and antidiabetic agents Cyclosporin ampicillin	https://clinicaltrials.gov/ct2/show/NCT04362332 https://clinicaltrials.gov/ct2/show/NCT04328493 https://clinicaltrials.gov/ct2/show/NCT04333628 https://clinicaltrials.gov/ct2/show/NCT04331600 https://clinicaltrials.gov/ct2/show/NCT04303507 https://clinicaltrials.gov/ct2/show/NCT04351191 https://clinicaltrials.gov/ct2/show/NCT04323527 https://clinicaltrials.gov/ct2/show/NCT04308668 https://clinicaltrials.gov/ct2/show/NCT04376814 https://clinicaltrials.gov/ct2/show/NCT04330690
Ivermectin			Abdominal pain, hypotension, mild ECG changes, peripheral and facial edema, transient tachycardia, hyperthermia, insomnia, somnolence, vertigo, pruritus, eosinophilia, leukopenia, elevated ALT/AST, myalgia, blurred vision, and Mazzotti reaction (with onchocerciasis)	Hypersensitivity to ivermectin	Warfarin	https://clinicaltrials.gov/ct2/show/NCT04360356 https://clinicaltrials.gov/ct2/show/NCT04351347 https://clinicaltrials.gov/ct2/show/NCT04374019
Azithromycin	Inhibits viral entry and endocytosis	Oral	QTc prolongation, diarrhea, nausea, and abdominal pain	Hypersensitivity to azithromycin, erythromycin, and any macrolides or ketolides History of cholestatic jaundice/hepatic dysfunction associated with prior use of azithromycin Long QT syndrome	Nelfinavir Warfarin Digoxin Colchicine Phenytoin	https://clinicaltrials.gov/ct2/show/NCT04359316 https://clinicaltrials.gov/ct2/show/NCT04332107 https://clinicaltrials.gov/ct2/show/NCT04336332 https://clinicaltrials.gov/ct2/show/NCT04341727
**Drugs Acting on Host Cell Receptors**						
Angiotensin-converting enzyme inhibitors	Increases ACE2 epithelial cell lung expression	Oral	Cough Creatinine increased Syncope Hyperkalemia Hypotension Diarrhea Chest pain Abdominal pain Rash Infection Asthenia Angina pectoris Dyspnea Pruritus Headache Dizziness Increased BUN and serum creatinine	Hypersensitivity to ACE inhibitors History of ACE inhibitor-induced angioedema and hereditary or idiopathic angioedema Coadministration of neprilysin inhibitors with aliskiren in patients with diabetes mellitus or with renal impairment	Salt substitutes with potassium Non-steroidal anti-inflammatory drugs Digoxin (only with captopril) Probenecid (only with captopril)	https://clinicaltrials.gov/ct2/show/NCT04366050 https://clinicaltrials.gov/ct2/show/NCT04355429 https://clinicaltrials.gov/ct2/show/NCT04345406 https://clinicaltrials.gov/ct2/show/NCT04374695
Angiotensin receptor blockers	Increases ACE2 epithelial cell lung expression	Oral	Dizziness Headache Hyperkalemia.	Bilateral renal artery stenosis	ACEi Aliskiren	https://clinicaltrials.gov/ct2/show/NCT04335123 https://clinicaltrials.gov/ct2/show/NCT04311177 https://clinicaltrials.gov/ct2/show/NCT04312009 https://clinicaltrials.gov/ct2/show/NCT04343001
Camostat	Inhibits TMPRSS2 and prevent viral-cell entry	Oral	Abnormal liver function tests, Diarrhea Hyperkalemia Itching Jaundice Low blood platelets Liver disorder GIT discomfort	Pregnancy (teratogenic)	None	https://clinicaltrials.gov/ct2/show/NCT04321096 https://clinicaltrials.gov/ct2/show/NCT04353284 https://clinicaltrials.gov/ct2/show/NCT04338906 https://clinicaltrials.gov/ct2/show/NCT04355052

Abbreviations: Abbreviations: ABCB: ATP Binding Cassette Subfamily B Member/ Multidrug Resistance Protein; ACEi: angiotensin converting enzyme inhibitor; ALT: alanine transaminase; AST: aspartate transaminase; AV: atrio-ventricular; BCRP/ABCG2: Breast cancer resistance protein (BCRP)/ATP-binding cassette subfamily G member 2 (ABCG2); CAD: coronary artery disease; CD: cluster of differentiation; CYP: cytochrome P450; DMARDs: disease modifying antirheumatic drugs; ECG: electrocardiography; G6PD: glucose 6-phosphate dehydrogenase; GIT: gastro-intestinal tract; IFN: interferon; Ig: Immunoglobulin; JAK: Janus kinase; OATP: organic anion transport protein; QTc: corrected QT-interval in the ECG; RdRp: RNA-dependent RNA polymerase enzyme; UGT1A: UDP- glucuronosyltransferase 1.
